# Exploring interprofessional collaboration in the intensive care unit

**DOI:** 10.4102/sajp.v80i1.2098

**Published:** 2024-11-11

**Authors:** Meluleki S. Thethwayo, Pat Camp, Diane van Staden, Verusia Chetty, Stacy Maddocks

**Affiliations:** 1 Discipline of Physiotherapy, School of Health Sciences, University of KwaZulu-Natal, Durban, South Africa; 2 Centre for Heart Lung Innovation, Faculty of Health Sciences, University of British Columbia, Vancouver, Canada; 3 Department of Physical Therapy, Faculty of Health Sciences, University of British Columbia, Vancouver, Canada; 4 Department of Nursing, Faculty of Health and Social Development, University of British Columbia, Okanagan, Canada

**Keywords:** intensive care, interprofessional collaboration, interprofessional education, clinicians, COVID-19, multidisciplinary team

## Abstract

**Background:**

Critical care units require an interprofessional management approach to optimise patients’ health. Clinical education and training delivered in remote healthcare settings are vital for fostering interprofessional collaboration (IPC) among health science students for future team functioning.

**Objectives:**

Our study explored the IPC among clinicians in the intensive care unit (ICU) setting at two South African decentralised clinical training facilities to understand the existing collaborative practices that students are exposed to during their clinical training.

**Method:**

A qualitative study design, utilising semi-structured interviews, was used to gather information on the experiences of 40 purposively selected participants working in the ICU settings at the two clinical sites. Data collected from the interviews were transcribed verbatim and thematically analysed.

**Results:**

Four major themes were identified from the data, namely, scope-of-practice dispute, teamwork disruption, organisational obstacles and future aspirations.

**Conclusion:**

Participants believed that a lack of professional regard by medical doctors and an inadequate understanding of the role of other professionals impeded appropriate referral practice and collaborative team functioning. Under-exposure to interprofessional education (IPE) at an undergraduate level and the pervasive medical hierarchy were perceived as a primary attributable cause of this phenomenon. Moreover, the coronavirus disease 2019 (COVID-19) pandemic and persistent staff shortages purportedly obstructed potential opportunities to collaborate in multidisciplinary meetings. Participants believed that improving undergraduate IPE and compulsory multidisciplinary meetings to promote communication would improve team functioning in these clinical settings.

**Clinical Implications:**

Undergraduate IPE is a feasible approach to improve collaborative care in ICUs to achieve better patient outcomes.

## Introduction

Intensive care unit (ICU) admission is often the beginning of a prolonged hospital stay for patients (Bhat, Vasanthan & Babu 2017; Morgan 2021; Donovan et al. 2018; Ervin et al. 2018). Their complex clinical needs necessitate a functional, collaborative relationship between clinicians in order to apply appropriate and timely interventions to promote the best outcomes (Costa et al. 2014; Mamo, Aklog & Gebremedhin 2023; Morgan 2021; Nygaard et al. 2023; Permanasari & Oktamianti 2023). Interprofessional collaboration (IPC) is the synergistic partnership between two or more healthcare professionals, communicating to solve problems, set goals and manage patient care to facilitate the best possible outcomes (Donovan et al. 2018; Nygaard et al. 2023). Good IPC facilitates appropriate referral practices in order to commence timely patient rehabilitation, recovery and discharge, which ultimately lowers medical costs while preserving medical resources (Costa et al. 2014; Donovan et al. 2018; Nørgaard et al. 2013; Permanasari & Oktamianti 2023; Rose 2011). Such outcomes are desirable for any healthcare setting but are crucial in resource-limited South African public hospitals (Govender et al. 2019; Malakoane et al. 2020).

Poor professional collaboration in ICUs is costly, compromises patient welfare and demonstrates poorer patient outcomes (Mamo et al. 2023; Matusov et al. 2022; O’ Connor et al. 2016; Rose 2011). For students completing professional healthcare training, poor collaborative clinical practice is associated with a lack of early interdisciplinary exposure, potentially jeopardising their future interprofessional cooperation (Bechard et al. 2010; Maddocks et al. 2018; Rose 2011; Stadick 2020). Undergraduate interprofessional education (IPE) has been regularly recognised as a feasible approach to enhance collaborative team functioning for improved patient assessment, planning, referral and outcomes, within the healthcare system (Karthikeyan & Jones 2015; Maharajan et al. 2017; Thistlethwaite 2012; Van Diggele et al. 2020).

In recent years, South African universities have initiated a clinical training strategy that aims to enhance opportunities for undergraduate interprofessional learning and collaboration through the introduction of decentralised clinical training (DCT) platforms. These geographically distant clinical sites are deemed to be dynamic environments to foster IPC where health science students can engage with the principles of IPE to learn from each other, with each other and about each other (Baloyi et al. 2020; Blose et al. 2019; Govender et al. 2018; Hansraj et al. 2022).

The ICUs at these sites are often without speciality care such as cardiac surgery, neurosurgery, among others and cater to a more generalised acute care patient population. Hence, the environment allows for simultaneous engagement with a potentially wider multidisciplinary group of professionals as opposed to specific groups of specialised professionals. Decentralised clinical training has reportedly fostered an improved interprofessional culture for students training at these facilities (Baloyi et al. 2020; De Villiers et al. 2017) and aims to produce health professionals with skills which may be aptly applied in undifferentiated patient populations (Baloyi et al. 2020; Gaede 2018). A substantial body of evidence exploring IPC has been conducted in other parts of the world; however, fewer studies have been conducted among resource-limited South African hospitals where final-year health science students receive their intensive care unit (ICU) training. Furthermore, the study was conducted during the coronavirus disease 2019 (COVID-19) pandemic, a time during which South African health system vulnerabilities were further accentuated (Liew et al. 2020; Xiang et al. 2020). The study aimed to explore the IPC among clinicians at selected DCT sites in order to understand the existing interprofessional practices that students are exposed to during their clinical training. The results of our study provide insight into the collaborative reforms to be considered by healthcare training institutions and professionals for improved IPC in South African public hospitals in the future.

## Research methods and design

### Design

Our study employed a phenomenological qualitative design, using semi-structured interviews with an interview guide consisting of open-ended questions which probed interprofessional communication, understanding of interprofessional roles, referral pathways, patient management planning practices and engagement with IPE. Furthermore, to explore IPC among clinicians in the ICU setting, to inform future undergraduate clinical education and collaborative clinical practice among participating healthcare professionals. Qualitative research allows the researchers to delve into the phenomenon, or the lived experiences of the healthcare workers and students who form part of the multidisciplinary team (Tenny, Brannan & Brannan 2023).

### Research setting

The study data were collected during the COVID-19 pandemic at two district-level public hospitals which, together with community centres, form part of the primary healthcare system where final-year health science students rotate to complete their compulsory clinical education. The institutions are in rural and peri-urban communities in the province of KwaZulu-Natal and admit patients with trauma, medical and general surgical conditions into their ICUs. The first institution had an eight-bedded ICU, and the second one a six-bedded ICU, located in the north coast and south coast of the KwaZulu-Natal province, respectively.

### Study population

The study population comprised of all clinicians (doctors; nurses; physiotherapists; dieticians; speech therapists and occupational therapists) who were working in the two ICU settings, as well as final-year physiotherapy students completing their compulsory clinical training in these units.

### Recruitment and sampling strategy

Heterogeneous purposive sampling was used (Palinkas et al. 2015) to select clinicians working in the ICU setting for a minimum of 3 months and final-year health science students rotating through the ICU. Varying age groups, genders, ethnicities and years of work experience were accounted for in the sampling strategy. Potential participants were recruited by face-to-face invitation on the ward.

### Data collection tools

An interview, guided by the literature (Gupte & Swaminathan 2016) and from discussions with an experienced ICU-trained health professional, was developed in order to elicit comprehensive information from the participants regarding their working relationships. The interview questions were reviewed to evaluate their suitability for the study’s aim by three academic qualitative researchers who were health professionals with experience working in these settings. Minor recommendations regarding the questions were suggested and were included in the final interview guide. The interview guide included open-ended questions that probed interprofessional communication, the understanding of professional roles, patient management planning, referral practices and engagement with IPE. Tape recorders were used for audio recordings of the subjects’ responses during the interviews. A pen and a notepad were used to record participants’ non-verbal cues.

### Procedure

Data was collected over 4 weeks. Ethics approval was obtained from the University of KwaZulu-Natal’s Research Ethics Committee (HSSREC/00001545/2020). Information sheets describing the purpose of and voluntary participation in the study, coupled with a written consent form to participate, were signed by the participants who met the inclusion criteria prior to the commencement of data collection. Semi-structured interviews were conducted in a pre-arranged private room at each hospital at the convenience of the participants. Interviews took approximately 45 min. During the interviews, two tape recorders were used to record the proceedings, and the interviewees’ reactions and non-verbal cues were observed and recorded in a notebook. The interviewing researcher was vigilant to adopt a neutral attitude while interviewing and exercised caution to avoid question-wording bias through leading questions. Data were collected until no new information emerged. All recorded data were stored electronically on password-protected electronic files. Data were transcribed verbatim following the interviews, and transcripts were prepared for analysis.

### Data analysis

**FIGURE 1:**
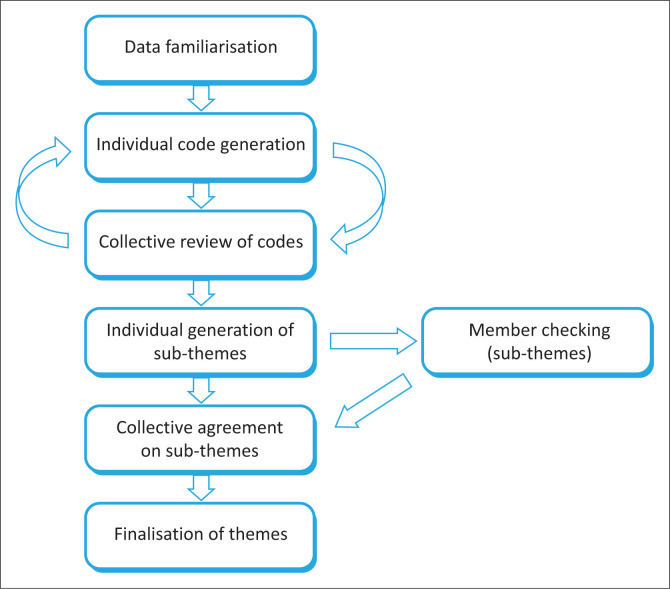
Data analysis process.

A step-wise approach (see Figure 1) to thematic data analysis was employed (familiarisation; coding; initial theme generation; theme review) to finalise themes (Braun & Clarke 2014). The transcribed data were read and re-read, first for familiarisation and to obtain a general impression of the overall data; and subsequently to identify codes and emergent themes through an inductive process. Trustworthiness was ensured by member-checking of quotes and themes with a random selection of participants in order to validate the findings, as well as through the use of thick, rich descriptions. Dependability was ensured through peer review of the researcher’s generated quotes and themes by research supervisors (S.M. and V.C.). Themes were discussed and disputed among the authors until agreement on the final themes to be included was reached through analyst triangulation.

Transferability was achieved through the authors’ detailed documentation of the study context, methodology and purpose. These techniques were employed to confirm that the results represent the participants’ experiences and not merely the researcher’s perceptions (Shenton 2004). Pseudonyms were used to report participants’ illustrative quotes and maintain anonymity.

### Ethical considerations

Ethics approval to conduct the study was obtained from the University of KwaZulu-Natal’s Research Ethics Committee (HSSREC/00001545/2020). All procedures were carried out in accordance with the approved relevant ethics guidelines. Written informed consent was obtained from each study participant after a clear explanation about the purpose of the study and the significance of their participation was explained. All participants were informed of their right to refuse to participate or withdraw at any time. Confidentiality of the information was assured by de-identification of audio files and transcripts which were password-protected; and pseudonyms were used in-text to maintain participant anonymity.

## Results

In all, 40 participants – medical doctors, nurses, physiotherapists, physiotherapy students, occupational therapists, speech therapists and dieticians – across all four South African ethnic groups participated in the study. The participants included 9 ICU doctors, 8 nurses, 6 physiotherapists, 2 speech therapists, 3 occupational therapists, 2 dieticians and 10 physiotherapy students. Eight of the participants were males, of which two were doctors, one was an occupational therapist and five were physiotherapy students. The clinicians’ working experience ranged from 2 months to 29 years in public healthcare practice. Table 1 reflects the participant demographics.

**TABLE 1:** Participant demographics (*N* = 40).

Variable	*n*
**Age (years)**	
21–25	18
26–30	4
31–35	4
36–40	3
> 40	11
**Gender**	
Female	32
Male	8
**Profession**	
Doctor	9
Nurse	8
Physiotherapist	6
Occupational therapist	3
Speech therapist	2
Dietician	2
Physiotherapy student	10

### Themes

The researchers identified four major themes from the data collected: scope-of-practice disputes; teamwork disruption; organisational obstacles and aspirations and recommendations for improved IPC. Table 2 reflects the themes, sub-themes and illustrative quotes from the interviews.

**TABLE 2:** Themes, sub-themes and participant quotations.

Themes	Sub-themes	Participant quotations
Scope-of-practice dispute	Perceived understanding of interdisciplinary professional roles	‘I am a fully trained ICU nurse specialist, but I don’t think some doctors understand that. I don’t think they understand my capabilities. I feel that there are limitations in doing my work’. (Nurse 1, female, 10 years’ experience) ‘Oh. It’s bad. It’s so bad that I actually had to find out exactly, during their training, especially the doctors, how well are they taught about the therapists’ role. It was actually just a ten-minute slide lecture: the physiotherapists do this, and this, and that and then let’s move along. In our institution, every year when we get new interns or new staff, we do the presentation [*to inform them*]. What is a physio? What do they do? How do you refer to the physio and where do you refer to the physiotherapist?’ (Physiotherapist 5, female, 15 years’ experience) ‘I feel, like, not many people in the ICU don’t understand what we do, especially doctors. I find that rehabilitation professionals [*are*] much more receptive of what we do’. (Speech therapist 1, female, 3 months’ experience) ‘We know, like, the basic things that, for example, what a dietician does, or a speech therapist. But we don’t know, like, the actual detail’. (Doctor 5, female, 9 months’ experience)
	Inappropriate and untimely referrals	‘They [*doctors*] refer the patient to physio, once they are done with the patient. We don’t treat the patient at the same time with the doctors. They only think of physio at the end, when they’re about to discharge the patient’. (Physiotherapist 2, female, 7 years’ experience) ‘Usually, we get inappropriate referrals like, getting a referral that basically says we must go play with children, whereas there is more to OT than play therapy’. (Occupational therapist 1, male, 3 years’ experience) ‘A lot of the times when I get to the referrals, it’s only close to when they get discharged, just so that they have everything ticked; but if I have to see the patient as soon as possible, then the outcomes might have been different’. (Speech therapist 2, female, 10 months’ experience)
	Education	‘I don’t think we have enough inter-professional education in our education system, so I think this is one aspect that needs to be definitely included or really emphasised in our curriculum’. (Doctor 1, female, 4 years’ experience) ‘It [*IPE*] is very important because it will also help us as clinicians, so that the patient does not deteriorate. Sometimes the patient deteriorates because of us as clinicians lacking interprofessional education. Right now, in the undergraduate level, student doctors see themselves as superior than anyone else [*referring to student doctors’ indifference toward the opinions of students from other disciplines]*’. (Final year physiotherapy student 5, male, no experience)
Teamwork disruption	Absence of interdisciplinary ward rounds	‘The physiotherapists, well, we don’t do rounds with them. They normally speak directly with a senior doctor … I feel like there’s room for improvement there’. (Doctor 3, female, 9 months’ experience) ‘I would say it [*teamwork*] is not that great because most of the time they won’t usually communicate with us directly. We don’t go as a collective to a patient’. (Doctor 4, female, 9 months’ experience) ‘Doctors will have their compulsory ward rounds alone. Nurses will have their compulsory ward rounds alone, the speech also and physio’. (Doctor 1, female, 4 years’ experience) ‘There are ward rounds. I can’t say people are forced, because not everybody does attend. If you want your instructions to be carried out, and if you want to be a valued member of the team, you have to show up – otherwise no one is going to take you seriously’. (Dietician 1, female, 10 years’ experience)
	Poor communication	‘I feel there is less communication … I feel that when a doctor makes changes, because I am always there, they should communicate with us’. (Nurse 1, female, 10 years’ experience) ‘As I said, the consultants, the doctors, they think they have the final word. They tend to think whatever they say goes until you argue with them’. (Nurse 5, female, 29 years’ experience) ‘If a surgeon comes and orders something [*without consulting the ICU doctor*] and the ICU doctor questions it afterward, then me as a nurse and as a medium of them all, I end up confused. Communication is poor’. (Nurse 4, female, 3 years’ experience)
	Impact of COVID-19	‘There was a shortage of staff and a lot of confusion with the allocation of staff. There were lots of people complaining’. (Doctor 6, male, 3 years’ experience) ‘It [*COVID-19*] has destroyed our teamwork. During the hard days of COVID-19, you’d come knowing that you would work in the ICU, but suddenly you were told you were going to work there [*COVID ward*] and there were conflicts’. (Nurse 1, female, 10 years’ experience) ‘It has challenged a lot, cause you find that some of the team members are sick. They are not at work and whoever are working, there is a lot of workload and some people, they get irritated like you are making me work more … There is shortage of doctors, shortages of physio, of speech because someone is sick. It just influenced workload in bad way’. (Doctor 2, female, 6 months’ experience)
Organisational obstacles	Shortage of staff	‘For now I think it’s just the shortage of personnel, the fact that we have a limited number of consultants in the different departments, so that makes it [*team ward rounds*] impossible because they’ve got other responsibilities’. (Doctor 1, female, 4 years’ experience) ‘It’s quite difficult to attend ward rounds, we being short-staffed. So, as from the therapists’ side, we rely more on the nurse for communication about the patient, and also on the file’. (Physiotherapist 5, female, 15 years’ experience)
	Financial burden	‘They [*hospital management*] will complain about the budget, especially with the allied professionals. They say allied falls under non-essential posts … They only prioritise nurses and doctors’. (Doctor 1, female, 4 years’ experience) ‘Each and every time we send a motivation to hire more staff, they don’t consider it. They will tell you about financial budget’. (Physiotherapist 5, female, 15 years’ experience)
Future Aspirations	Collaborative team practice to enhance understanding of professional roles and improved outcomes	‘I can’t over-emphasise that it [*teamwork*] is the most important thing in ICU, you know. I’m sure you know the theme for me: you cannot run ICU in isolation. It [*teamwork*] is going to affect [*lessen*] costs. It’s going to improve the well-being of the patient’. (Doctor 1, female, 4 years’ experience)
	Compulsory joint ward rounds to improve communication and referral practices	‘Communication in the form of interdepartmental meetings, ward rounds where everyone needs to be present. We must make time to communicate’. (Nurse 2, female, 20 years’ experience) ‘The ideal for me would be that rehab is included in ward rounds and discussing cases and discussing discharge planning, etc., and not only to be called at last minute; so to be involved with the process from the start’. (Speech therapist 2, female, 9 months’ experience)
	Interprofessional education	‘IPE, it’s the only way we can learn to communicate, from an undergraduate level. Maybe we can have a programme where we are tasked to do a home visit together so that we can see the different roles … ’ (Final year, male, physiotherapy student 6)

COVID-19, coronavirus disease 2019; ICU, intensive care unit; IPE, interprofessional education; OT, occupational therapist.

#### Scope-of-practice disputes

The theme, ‘scope-of-practice disputes’, and the sub-theme, ‘poor appreciation for breadth and boundaries of others’ clinical practice’ described the lack of understanding among doctors regarding the roles and functions of other team members. Nurses, physiotherapists, speech therapists and occupational therapists were united in declaring that the doctors lacked an understanding of, and appreciation for, their professional roles:

‘I feel, like, not many people in the ICU understand what we do, especially doctors. I find that rehabilitation professionals [*are*] much more receptive of what we do.’ (Speech therapist 2, female, 3 months’ experience)

Some of the participating doctors agreed with these sentiments, admitting that they had inadequate knowledge regarding the specifics of the scope of practice of other team members:

‘We know, like, the basic things that, for example, what, a dietician does, or a speech therapist. But we don’t know, like, the actual detail’ (Doctor 3, female, 9 months’ experience).

Consequently, referrals to the appropriate discipline-specific rehabilitation professionals were poorly planned and ill-timed.

#### Teamwork disruption

The second theme, ‘teamwork disruption’, was an overarching theme that was identified from quotes that described poor collaborative engagement among the clinicians. The doctors expressed their dissatisfaction with other health professionals, conducting ward rounds in silos and failing to communicate with them directly:

‘I would say it [*teamwork*] is not that great because most of the time they won’t usually communicate with us directly. We don’t go as a collective to a patient’ (Doctor 3, female, 9 months’ experience).

The doctor’s sentiments were criticised by the nurses and other rehabilitation professionals (excluding the dieticians), who believed that the doctors lacked regard for their professional contributions and dismissed them through a lack of communication in clinical care and decision-making:

‘I feel there is less communication … the doctor will see the patient, write something in the notes, and make some changes – sometimes not even telling you. Then when you’re doing a report, only then you will see that something was written [*ordered*]. I feel that when a doctor makes changes, since I am always there [*when he/she does it*], they should communicate with me.’ (Senior nurse 1, female, 10 years’ experience)

Clinicians also felt that COVID-19 further complicated teamwork dynamics as staff absenteeism was high and conflicts arose regarding the allocation of duties and responsibilities in the COVID-19 wards:

‘There was a shortage of staff [*during the pandemic*] and a lot of confusion with the allocation of staff. There were lots of people [*staff*] complaining’ (Doctor 6, male, 3 years’ experience)

#### Organisational obstacles

The third theme, ‘organisational obstacles’, highlighted the challenges around the shortage of staff and institutional financial limitations. Rehabilitation professionals reported that they often missed joint clinical ward rounds because of the scarcity of staff on the ground in relation to the workload irrespective of the pandemic. Furthermore, fiscal restrictions inhibited the opening of posts and the recruitment of new healthcare workers, particularly rehabilitation staff, as they were believed to be non-essential staff:

‘Each and every time we send a motivation to hire more staff, they don’t consider it. They will tell you about the financial budget. They say physios fall under non-essential posts.’ (Senior physiotherapist 5, female, 15 years’ experience)

#### Aspirations and recommendations for improved interprofessional collaboration

Finally, the theme ‘aspirations and recommendations for IPC’ described participants’ hopes for improved team practice. All the respondents explicitly asserted that teamwork was fundamental in the ICU for improved patient outcomes. Compulsory joint ward rounds and interdisciplinary meetings, where all professionals participate in clinical decision-making, were recommended as a strategy to improve communication and collaborative team practice. Furthermore, participants believed that IPE should be more deliberately integrated into undergraduate classrooms and clinical practice to improve early understanding of interdisciplinary roles, collaborative team practice and communication in the ICU.

## Discussion

The delivery of quality care in any ICU is contingent on an interprofessional collaborative approach and has considerable meaning in South Africa’s public sector, where service provision is challenged by significant resource limitations (De Beer, Brysiewicz & Bhengu 2011; Mamo et al. 2023; Permanasari & Oktamianti 2023). Rural acute care environments are prime potential spaces for fostering IPC (Alexanian et al. 2015; Mamo et al. 2023; Permanasari & Oktamianti 2023) and the chosen study settings typify the level of public sector care provision accessed by a large number of South Africans. This paper explored perceptions of existing IPC between clinicians at two South African decentralised clinical training ICU settings.

The range of work experience among the participants in our study was wide, but opinions expressed by members of the various professional groups regarding their interprofessional encounters were largely uniform. Both nurses and rehabilitation professionals believed that doctors demonstrated a limited understanding of their professional roles and capabilities, which belittled their value. The doctors’ poor insight was perceived as disregarding and undermining of other healthcare professionals’ contributions to the team. These sentiments were similar to the findings of South African studies, where poor understanding of interdisciplinary roles among the clinicians was viewed as a common barrier towards achieving collaborative practice in the hospital setting (Crafford et al. 2023; Maddocks et al. 2018; Mokoena, Rabie & Du Preez 2023). The importance of acknowledging and upholding professional distinctiveness or identity among health professionals improves their resilience and confidence, which promotes contribution, team cohesion and improved outcomes (Fitzgerald 2020; Mak et al. 2022). Rehabilitation professionals raised concerns regarding the doctors’ referral practices and believed that referrals were often a ‘tick-box exercise’ completed just prior to discharging a patient from the hospital and often lacking in sound clinical judgement. These acknowledgements were demotivating for rehabilitation staff and negatively influenced their attitude towards team cooperation. This medical hierarchy described by participants has been cited as an important reason for professional division, team dysfunction and poor referral practices in other studies (Chetty & Maharaj 2013; Ntinga & Van Aswegen 2020; Rogers, De Brún & McAuliffe 2023). The perception of poor partnership and coordination of care among the health professionals revealed in this study raised questions regarding the quality of treatment provided for these ICU patients in the study settings (Morgan 2021; Thomas 2011).

In contrast to the views of the nurses and rehabilitation professionals, the ICU doctors (intensivists) in our study believed that diminished team efficacy was primarily because of poor interprofessional communication caused by the low attendance of other professionals at interdisciplinary ward rounds. One health professional in our study, who diligently attended ward rounds, concurred with the doctors’ sentiments, highlighting that the absence of rehabilitation professionals at multidisciplinary ward rounds limits their opportunity for contribution, collaborative planning and joint patient progress monitoring. The results of recent qualitative studies among health professionals at hospitals both in South Africa and internationally indicated similar findings to our study (Kock et al. 2021; Walton et al. 2019), where low attendance of rehabilitation professionals at interdisciplinary meetings was reportedly because of time constraints from competing clinical demands and low motivation to contribute as a result of feeling undervalued (Zamanzadeh et al. 2021).

Although it is understandable that staff shortages may hamper the availability of all team members at a meeting, the apparent poor communication that existed between the health professionals in our study is improper, particularly in an acute care academic setting and during a global pandemic. Interprofessional contributions in joint clinical meetings, particularly in the ICU, should be regarded as a compulsory component of acute care, as failure to communicate among clinicians is one of the most frequently cited reasons for poor patient outcomes (Aparanji et al. 2018; Matusov et al. 2022; Ntinga & Van Aswegen 2020). The team culture described in our study, while not unique, is a legitimate concern for participating students in training at these clinical placements. A study that explored health professional students’ observations about interprofessional collaborative practice during rural clinical placements found that, where the medical hierarchy was less rigid, with more and interprofessional respect, teamwork was more efficacious (Carney et al. 2021).

Boltey et al. (2023) describe 10 enablers of IPC in an ICU and explain how efforts from both the doctors and other team members are essential for collaborative practice to occur. The authors describe how tenets like the validation of, and invitation to, other health professionals from physicians, coupled with reciprocal active engagement on ward rounds by all participants, strengthen IPC.

The participants’ perceptions of team functioning in our study were in stark contrast to the reported potential of IPC to expand a team’s clinical capacity in remote settings (Permanasari & Oktamianti 2023; Spencer et al. 2015). Recent studies have explained that, although teamwork in an ICU may not always look like the idealised concept of togetherness, it should still reflect collaboration, networking and coordination (Alexanian et al. 2015; Permanasari & Oktamianti 2023). These attributes were largely missing from the study results.

Students rotating through these clinical sites were discouraged by their observations of team functioning in the ICU and, together with the health professionals, recommended that interprofessional collaborative management of patients among students be more deliberately integrated into their clinical training programme in order to prepare them for interprofessional practice after graduation. In their South African studies, Ntinga and Van Aswegen (2020) highlighted similar sentiments, promoting IPE to enhance IPC among clinicians in the hospital setting. Walker, Barnett and Cross (2021) noted that an implicit assumption exists that clinicians are well-placed to provide IPE. However, many of them have not received this training either. A promising example of the potential for IPE to enhance patient care was demonstrated by Schmid et al. (2022), who showed that targeted IPE regarding antibiotic administration among nurses, physicians, and pharmacists at one institution resulted in the optimisation of antibiotic therapy over a 7-year period. Interprofessional education, delivered in a clinical environment is considered the ideal platform for IPC application, where the appreciation of interdisciplinary functions and collaborative role modelling can be observed in real work settings rather than just theoretically in the classroom (D’Eon et al. 2010; Maree & Van Wyk 2016; Ntinga & Van Aswegen 2020; Stadick 2020; Van Diggle et al. 2020). Both students and healthcare professionals agreed on the perceived value of learning about each other’s roles through consultation as a collective.

The World Health Organization (WHO) Framework for Action on Interprofessional Education and Collaborative Practice (2010) emphasised the value of IPE in strengthening global health systems and accentuated the need for contextualising the appropriate mechanisms in different settings. Missen et al. (2012) explored the factors that promote IPE learning and practice in rural contexts and highlighted that an explicit IPE agenda in interdisciplinary undergraduate curricula, coupled with organisational readiness for IPE and a singular message communicated to all key stakeholders about the plan and purpose for IPE, is a good place to start to garner the appropriate support required. The authors further suggested that IPE learning objectives be specifically prescribed by the disciplinary education providers and actively promoted by regulatory bodies.

Furthermore, Walker, Cross and Barnett (2019) recommended the identification of local interprofessional learning opportunities (e.g., multi-professional student team patient consultation, as suggested by participants in our study), the identification of IPE leaders and/or champions within the healthcare institutions to facilitate the organisation, and the development of activities and opportunities for students to share their meaningful IPE experiences with other disciplines. These recommendations are sound, achievable ideas for decentralised clinical settings to work towards. Studies exploring the experience and perceptions of IPE in other rural contexts have demonstrated positive learning outcomes for participating students (Stadick 2020; Van Diggele et al. 2020; Walker et al. 2019).

### Limitations

Our study was conducted at the clinical education sites of a single tertiary institution in one province. Therefore, caution should be observed in generalising the findings. However, the lessons learned are widely applicable. A further limitation is that the views of pharmacists were not explored in this review as they are not present within these ICUs on a regular basis, and were not available for interviews.

The interview guide, with open-ended questions, was evaluated by health professionals for suitability of administration, but was not piloted before data collection began.

## Conclusion

A unique aspect of our study was the exploration of IPC in a setting that was both decentralised and acute – contexts believed to be equally ideal for the fostering of IPC; yet the potential of this strategy to strengthen healthcare delivery in a resource-limited setting was not actualised. Interprofessional collaboration in the ICUs at these remote clinical training sites was perceived to be hampered by familiar issues, such as poor understanding of the scope of practice and the medical hierarchy. In addition, staff shortages and challenges related to the COVID-19 pandemic complicated team functioning still further, and these challenges were difficult to overcome. Participants believed that, at the very least, efforts to develop appropriate IPE delivery, improvements in interprofessional respect and participation in scheduled clinical meetings, were essential to improve the application of IPC in decentralised ICU settings.

Although our study was conducted in a developing world context, the underlying challenges are comparable to other developed world contexts and explain how poor collaboration affects care delivery. While it may be beyond our ability to change staffing complements to improve service delivery in acute care units, academics and health professionals alike are urged to think more creatively about the delivery of IPE in these settings. Further studies should explore how an intended IPE programme in clinical sites like these will influence student and health professional perspectives and practice of IPC in the future.
